# Commentary “The sexualized-body-inversion hypothesis revisited: Valid indicator of sexual objectification or methodological artifact?”

**DOI:** 10.3389/fpsyg.2015.00845

**Published:** 2015-06-18

**Authors:** Philippe Bernard, Sarah J. Gervais, Jill Allen, Olivier Klein

**Affiliations:** ^1^Department of Psychological Sciences, Center for Social and Cultural Psychology, Université Libre de BruxellesBrussels, Belgium; ^2^Department of Psychology, University of Nebraska-LincolnLincoln, NE, USA; ^3^Department of Psychology, Montana State UniversityBozeman, MT, USA

**Keywords:** sexual objectification, inversion effect, dehumanization, analytic processing, gender

Recent objectification research found results consistent with the sexualized body-inversion hypothesis (SBIH): People relied on analytic, “object-like” processing when recognizing sexualized female bodies and on configural processing when recognizing sexualized male bodies (Bernard et al., 2012). Specifically, Bernard et al. (2012) showed that perceivers were better at recognizing sexualized male bodies when the bodies were presented upright than upside down, whereas this pattern did not emerge for sexualized female bodies; thus, male bodies were recognized configurally similar to other human stimuli whereas female bodies were recognized analytically, similarly to most objects (see Kostic, 2013 for an exact replication). Based on two studies, Schmidt and Kistemaker (2015) concluded that Bernard et al. (2012)'s findings were: (i) due to a symmetry confound; (ii) not due to target's sexualization. This commentary challenges these conclusions.

## Stimulus set-up and symmetry confounds

In the sexualized body-inversion task, symmetry can impair the recognition of stimuli and this impairment is amplified when analytical (vs. configural) processing becomes more important (i.e., recognition of inverted bodies). Because Bernard et al. (2012) presented half of the stimuli upright and the other half inverted, Schmidt and Kistemaker suggest that Bernard et al.'s findings might be due to less stimulus symmetry among inverted (vs. upright) female bodies: If inverted female bodies are less symmetrical than upright female bodies in Bernard et al.'s setup, their findings could be explained by inverted (vs. upright) female bodies being simply easier to recognize than inverted (vs. upright) males bodies, rather than because of stimulus gender.

In their first study, Schmidt and Kistemaker examined symmetry in Bernard's stimuli and found that female bodies were more asymmetrical than male bodies. Strikingly, neither the interaction between stimulus gender and stimulus orientation nor the three-way interaction involving stimulus set-up emerged. These results suggest that differences in symmetry between inverted (vs. upright) female bodies (vs. male bodies) are not more pronounced in the Bernard set-up than in the counterbalanced set-up.

In their second study, these authors replicated Bernard et al. (2012)'s findings with Bernard's stimulus set-up, but they did not replicate the critical interaction between stimulus gender and stimulus orientation when presenting the stimuli in both positions. The authors concluded that Bernard et al.'s results were due to symmetry confounds with stimulus subsets (p. 83). However, like these authors, Bernard et al. (2015) presented all of Bernard et al. (2012)'s stimuli in both positions and successfully replicated the critical interaction (and they also conceptually replicated this pattern across two other experiments).

Schmidt and Kistemaker identified a potential stimulus set-up confound in their second study (contrary to Bernard et al., 2015), but their results are nonetheless inconsistent with the notion that symmetry could explain the pattern of results of Bernard et al. (2012). If a symmetry confound existed as Schmidt and Kistemaker suggest, symmetry should impair recognition scores more when analytic (vs. configural) processing becomes more important, with a greater inversion effect for “symmetrical” female bodies than for “asymmetrical” female bodies (Schmidt and Kistemaker, 2015, p. 78). We consider two robust tests –not reported by Schmidt and Kistemaker– that *directly* address this putative symmetry confound as they allow comparison *within* symmetry-matched stimuli (i.e., bodies presented in *both* positions). A visual inspection of Figure 2 (Schmidt and Kistemaker, 2015, p. 80) shows (i) a similar inversion effect emerged for *both* symmetrical (third vs. eighth bar) and asymmetrical female bodies (seventh vs. fourth bar); (ii) asymmetrical inverted female bodies (fourth bar) were *not* recognized better than symmetrical inverted female bodies (eighth bar).

In sum, contrary to Bernard et al. (2015), Schmidt and Kistemaker did find a stimulus setup effect, which suggests that future research is needed to explain these conflicting findings. However, based on Schmidt and Kistemaker's data, it is unlikely these different findings can be explained by a subset-symmetry confound.

## Role of target sexualization

Schmidt and Kistemaker also found an inversion effect for male and female bodies and this pattern occurred for naked bodies with or without masked sexual body parts. They concluded that Bernard et al.'s findings are not driven by target sexualization (Schmidt and Kistemaker, 2015, p. 84). We suggest that this conclusion is problematic and thus does not undermine the SBIH (Bernard et al., 2012).

First, although informative with regard to the role of target sexualization and inversion, the SBIH was posited to explain differences in recognition of sexualized male vs. sexualized female bodies (i.e., stimulus gender effect: Bernard et al., 2012, 2013), so it remains unclear how these findings weaken Bernard et al.'s original hypothesis regarding the moderating role of stimulus gender. Second, Schmidt and Kistemaker showed that naked stimuli (with and without a mask) were processed configurally, regardless of stimulus gender. But is the latter result informative regarding the role of target sexualization in Bernard et al. (2012)'s findings? From a *conceptual* replication perspective, Schmidt and Kistemaker provide evidence in favor of restricted generalizability of Bernard et al.'s findings but from a *direct* replication perspective Schmidt and Kistemaker's paper cannot address the role of target sexualization in Bernard et al.'s stimuli because they did not manipulate sexualization of these stimuli. Bernard et al. (2015), however, addressed this question and showed that an inversion effect emerged when sexual body parts (e.g., breasts) were less salient (i.e., pixelated) whereas this was not the case when non-sexual body parts (e.g., arms) were less salient, suggesting that the analytic processing of sexualized female bodies was due, in part, to a focus on sexual body parts.

In sum, contrary to Bernard et al. (2015), Schmidt and Kistemaker (2015) did find a stimulus setup effect but they did not offer compelling evidence that symmetry explained the results of Bernard et al. (2012). Consequently, exact replication studies with larger samples are needed to assess the SBIH, and we recommend statistically controlling for body symmetry while performing the same tests as in Bernard et al. (2012). Finally, we invite future research to further address important moderators of the SBIH, such as the role of target sexualization.

### Conflict of interest statement

The authors declare that the research was conducted in the absence of any commercial or financial relationships that could be construed as a potential conflict of interest.
